# Differences in urinary biomarker profiles in patients with nephrotic-range proteinuria with and without hypoalbuminemia: a single-center cross-sectional study

**DOI:** 10.3389/fendo.2026.1938326

**Published:** 2026-10-02

**Authors:** Mohan Liu, Yingnan Liang, Zhijia Zhang, Jiajia Bai, Zhenjie Chen, Jingwei Zhou

**Affiliations:** 1 Department of Nephrology, Dongzhimen Hospital, Beijing University of Chinese Medicine, Beijing, China; 2 Department of Nephrology, Hohhot Hospital of Traditional Chinese Medicine and Mongolian Medicine, Hohhot, China

**Keywords:** chronic kidney disease, diabetic kidney disease, megalin, nephrotic-range proteinuria, tubular injury

## Abstract

**Background:**

Patients with chronic kidney disease (CKD) presenting as nephrotic-range proteinuria (NR, ≥3.5g/d) exhibit distinct clinical phenotypes with and without hypoalbuminemia. The underlying mechanisms need further investigation.

**Methods:**

This retrospective study included patients with CKD hospitalized over the past two years with baseline 24-hour urine total protein (24-hUTP) exceeding 3.5g. Based on whether their baseline serum albumin (ALB) levels were ≤30 g/L, patients were assigned to two groups: the nephrotic syndrome (NS) group and the isolated nephropathy-range proteinuria (isolated NR) group. Clinical indicators and urine tubular injury biomarkers were compared between the two groups. The Spearman correlation coefficient was used to analyze the association between clinical indicators and urinary biomarker profiles. Urinary biomarkers and the isolated NR phenotype were evaluated for their independent association using multivariate logistic regression analysis.

**Results:**

In all, 134 patients were divided into 83 isolated NR and 51 NS groups. Urinary transferrin (uTRF) and N-acetyl-β-D-glucosaminidase (uNAG) were higher in the NS group than in the isolated NR group. The isolated NR group, in contrast, had significant high level of urinary retinol-binding protein (uRBP). Additionally, the two groups’ uRBP exhibited distinct correlation patterns with proteinuria. Multivariate logistic regression study demonstrated that uRBP and uTRF were independently linked to the phenotypes after controlling for relevant variables.

**Conclusions:**

Patients with NR with and without hypoalbuminemia exhibited distinct urinary biomarker profiles. Higher uRBP levels in patients without hypoalbuminemia may indicate differences in proximal tubular handling of low-molecular-weight proteins. In contrast, higher uTRF and uNAG levels in patients with hypoalbuminemia may reflect greater glomerular permeability abnormalities accompanied by tubular cellular injury. These findings are hypothesis-generating, and the potential involvement of megalin-mediated protein handling requires direct experimental and histopathological validation.

## 1 Introduction

The Global Burden of Disease study (GBD) estimates that 788 million people worldwide suffered from chronic kidney disease (CKD) in 2023. Compared to 1990, the age-standardized prevalence of CKD in adults worldwide rose by 3.5%, making it the ninth most common cause of death worldwide and responsible for 11.5% of deaths from cardiovascular disease (1). Proteinuria is an essential indicator of the advancement of CKD and is closely linked to an increased risk of end-stage renal disease (ESRD) (2). Nephrotic-range proteinuria (NR) patients also have a markedly elevated risk of cardiovascular events (3, 4). The etiology and clinical phenotype of CKD with NR are complexly heterogeneous. Although the majority of NR patients have hypoalbuminemia, some cases of diabetic kidney disease (DKD), membranous nephropathy (MN), and IgA nephropathy (IgAN) present with NR while maintaining serum albumin (ALB) levels >30 g/L (5–7). The underlying mechanisms have yet to be fully understood.

Damage to the glomerular filtration barrier is the main cause of NR. However, the role of compromised tubular reabsorption function has also received a lot of attention lately (8). Physiologically, the proximal tubule (PT) reabsorbs filtered proteins via endocytic receptors such as megalin and cubilin, followed by lysosomal hydrolysis. Reduced expression or dysfunction of megalin may lead to decreased reabsorption of ligands, such as retinol binding protein (RBP) and α1-microglobulin (AMG), and consequently contribute to increased urinary protein excretion (9). Therefore, alterations in megalin expression or function may represent a key mechanism explaining the preservation of ALB in patients presenting with NR without hypoalbuminemia.

Renal tubular tissue damage and dysfunction can be detected by urine biomarkers such as RBP, AMG, and N-acetyl-β-D-glucosaminidase (NAG). They have become important research tools since they are strongly associated with CKD progression and declining kidney function (10–12). Therefore, we performed a retrospective analysis on patients in our department who presented with NR. Based on their baseline ALB levels, individuals with nephrotic syndrome (NS) (ALB ≤ 30 g/L) and nephrotic-range proteinuria (NR) (ALB>30 g/L) were separated into two groups. By comparing differences in tubular injury markers between these two groups, we hope to investigate the relationship between urine biomarkers and the isolated NR phenotype, as well as provide potential mechanistic ideas.

## 2 Materials and methods

### 2.1 Patients

This retrospective analysis comprised CKD patients who were admitted to Beijing University of Chinese Medicine’s Dongzhimen Hospital’s Nephrology Department between December 2023 and December 2025, with a baseline 24-hour urine protein quantification (24-hUTP) of ≥3.5g. The diagnostic criteria for CKD referred to the KDIGO 2024 Clinical Practice Guideline for the Evaluation and Management of Chronic Kidney Disease (13). The diagnostic criteria for DKD was based on the 2024 American Diabetes Association (ADA) guidelines for the management of diabetic chronic kidney disease (14). Kidney biopsy was the basis for diagnosing MN, IgAN, and other conditions. Exclusion Criteria: Patients undergoing renal replacement therapy or kidney transplantation; Individuals with active infections that include hepatitis, tuberculosis, syphilis, or HIV/AIDS; Those with acute kidney injury (AKI) or a history of malignancy.

Patients were categorized into the nephrotic syndrome (NS) group and the isolated nephropathy-range proteinuria (isolated NR) group based on whether their baseline ALB levels were ≤30 g/L.

Subgroup analyses stratified by etiology were pre-specified in the statistical analysis plan to assess whether the association between urinary biomarkers and the isolated NR phenotype is influenced by the type of kidney disease. The subgroup results should be viewed cautiously due to the exploratory character of these analyzes.

This research protocol complies with the principles of the Declaration of Helsinki and has been approved by the Ethics Committee of Dongzhimen Hospital, Beijing University of Chinese Medicine (Approval number 2023DZMEC-598-02).

### 2.2 Clinical information and urinary biomarkers

For each participant, the following clinical and demographic information was gathered: age, sex, and primary kidney disease. Laboratory parameters included 24-hUTP, ALB, creatinine (Scr), hemoglobin (Hb), low-density lipoprotein (LDL), total cholesterol (TC), urea, and uric acid (UA).

Urinary transferrin (uTRF) was assessed as a marker primarily reflecting altered glomerular permeability. Urinary NAG (uNAG), a lysosomal enzyme released from proximal tubular epithelial cells (PTECs), was used as a marker of tubular cellular injury. Urinary RBP (uRBP) and AMG (uAMG), both low-molecular-weight proteins that are normally filtered and subsequently reabsorbed by the PT, were assessed as markers reflecting PT protein handling and reabsorptive function. All clinical and laboratory data were extracted from the initial medical records. We calculated the estimated glomerular filtration rate (eGFR) using the CKD-EPI formula.

### 2.3 Statistical analysis

R software version 4.5.2 was used for statistical analysis. *P*-values below 0.05 were deemed statistically significant. To determine normality, the Shapiro-Wilk test was employed. The mean ± standard deviation (SD) is the expression for continuous data that follows a normal distribution. In contrast, the median with an interquartile range (IQR) is used to represent those with a non-normal distribution. Categorical data are described using counts (percentages). For regularly distributed data, the independent samples t-test was employed to compare continuous variables between two groups; for non-normally distributed data, the Wilcoxon rank-sum test was employed. To compare categorical variables between groups, the chi-square test was employed. Correlations between urinary biomarkers and clinical parameters were analyzed using Spearman’s correlation analysis, and the Benjamini-Hochberg method was used to reduce the false discovery rate (FDR). The association between urinary biomarkers and the isolated NR phenotype was analyzed using multivariate logistic regression, adjusted for relevant clinical factors; missing data were handled using chained equation multiple imputation, and urinary biomarker concentrations were expressed as the urine-to-creatinine ratio. The Supplementary Materials contain comprehensive statistical techniques.

## 3 Results

### 3.1 Characteristics of patients with CKD

The final analysis comprised 134 individuals with NR. As outlined in **Table 1**, the cohort consisted of 77 (57.46%) patients with DKD, 46 (34.33%) with chronic glomerulonephritis (CGN), and 11 (8.21%) with other or unknown etiologies. Of these,83 (61.9%) patients were classified into the isolated NR group, with a median ALB of 35.60 g/L(IQR:33.00, 38.45);51 (38.1%) patients were classified into the NS group, with a median ALB of 25.50 g/L(IQR:22.00, 28.25). In comparison to the isolated NR group, the NS group showed noticeably higher levels of TC, LDL, and 24-hUTP. Conversely, there were statistically significant differences (*P* < 0.05) between the isolated NR and NS groups in terms of Scr and urea levels. The two groups did not differ statistically significantly in terms of age, Hb, or UA levels.

**Table 1:** Baseline characteristic comparison of CKD patients.

Characteristic	Overall (n=134)	Isolated NR (n=83)	NS (n=51)	*P* value
Male sex(%)	96 (71.64)	68 (81.93)	28 (54.90)	0.002
Age(mean (SD)), yr	54.16 (14.18)	54.66 (13.01)	53.35 (16.02)	0.606
Hemoglobin(mean (SD)), g/L	115.52 (21.62)	114.47 (20.78)	117.24 (23.03)	0.474
Creatinine(median [IQR]), μmol/L	244.55 [103.82, 357.68]	282.30 [134.35, 449.55]	141.80 [88.40, 244.55]	<0.001
eGFR (median [IQR]),mL/min/1.73 m^2^	26.47 [16.28, 67.75]	20.69 [11.86, 47.32]	45.88 [21.43, 82.54]	<0.001
Urea(median [IQR]), mmol/L	13.89 [7.87, 20.49]	16.55 [9.81, 23.74]	10.25 [6.57, 15.13]	<0.001
Uric acid(mean (SD)), μmol/L	407.97 (98.98)	420.08 (100.55)	388.26 (94.02)	0.071
Serum albumin(median [IQR]), g/L	31.80 [27.33, 36.98]	35.60 [33.00, 38.45]	25.50 [22.00, 28.25]	<0.001
24hUTP(median [IQR]), mg	6504.00 [4871.75, 9227.75]	5862.00 [4453.00, 8517.50]	8338.00 [6010.00, 11268.50]	<0.001
Total cholesterol (median [IQR]), mmol/L	4.94 [4.07, 6.53]	4.40 [3.65, 5.36]	6.16 [4.97, 7.91]	<0.001
Low density lipoprotein (median [IQR]), mmol/L	2.80 [2.23, 4.12]	2.41 [1.90, 3.13]	3.73 [2.71, 5.10]	<0.001
Primary disease(%)				
Diabetic kidney disease	77 (57.46)	60 (72.29)	17 (33.33)	<0.001
Chronic glomerulonephritis	46 (34.33)	16 (19.28)	30 (58.82)	
Other or unknown	11 (8.21)	7 (8.43)	4 (7.84)	

### 3.2 Comparison of urinary RBP, TRF, NAG, and AMG levels between the two groups

In comparison to the isolated NR group, the NS group had noticeably greater levels of uNAG (3.56[2.11, 5.46] U/mmol) and uTRF (15.42[10.63, 25.40] mg/mmol), as seen in **Figure 1**. Conversely, the uRBP levels in the isolated NR group were noticeably higher (5.81[2.62, 8.60] mg/mmol). (all *P* < 0.05).

**Figure 1:**
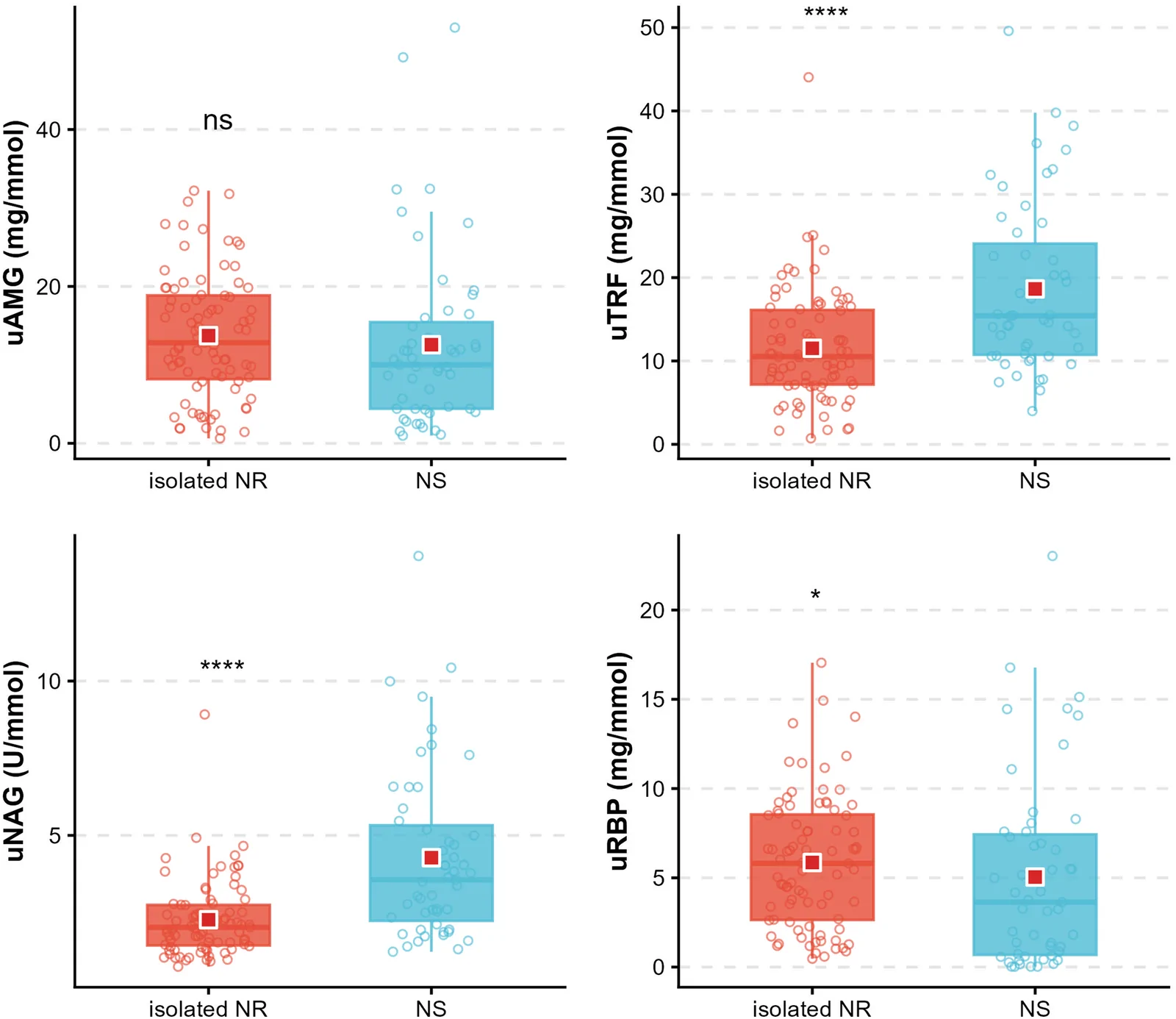
Comparison of urinary biomarker levels between the two groups. The NS group showed significantly higher levels of uNAG and uTRF than the isolated NR group. On the other hand, the isolated NR group’s uRBP levels were much greater. Isolated NR, the isolated nephrotic-range proteinuria group; NS, the nephrotic syndrome group; uAMG, urinary α1-microglobulin; uTRF, urinary transferrin; uRBP, urinary retinol binding protein; uNAG, urinary N-acetyl-β-D-glucosaminidase; (*p < 0.05, **p < 0.01, ***p < 0.001,****p < 0.0001, ns, *p* > 0.05.)

### 3.3 Correlation between urinary biomarkers and other clinical indicators

As shown in **Figure 2**, the Benjamini-Hochberg method was used to perform FDR correction on all correlation test results. The corrected results showed that uTRF and uNAG showed a positive correlation with 24-hUTP (r = 0.61, P < 0.001; and r = 0.37, P < 0.01, respectively) and a negative correlation with ALB (r = -0.47, P < 0.001; and r = -0.52, P < 0.001, respectively). Additionally, there was a negative correlation between the eGFR and uAMG and uRBP (r = -0.77, P < 0.001; and r = -0.33, P < 0.05, respectively). Furthermore, uNAG showed positive correlations with LDL(r = 0.42, P < 0.001) and TC (r = 0.41, P < 0.001).

**Figure 2:**
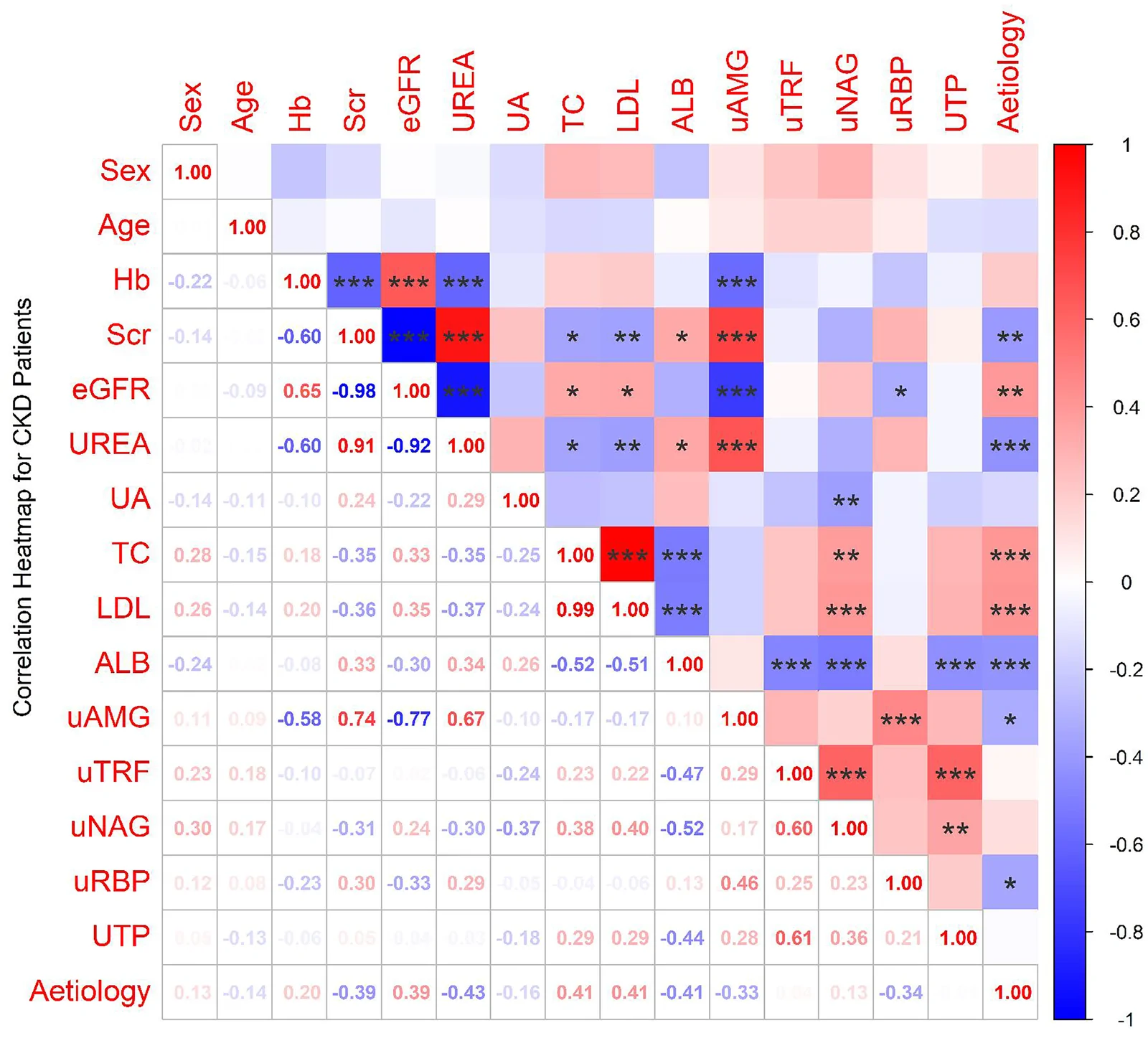
Correlation heatmap between urinary biomarkers and other clinical indicators. uTRF and uNAG exhibited a negative connection with ALB and a positive correlation with 24-hUTP. The eGFR was negatively correlated with uAMG and uRBP. Additionally, the TC and LDL were positively correlated with uNAG. Hb: Hemoglobin; Scr: Serum Creatinine; eGFR: Estimated glomerular filtration rate; UREA: urea; UA: Uric acid; TC: Total cholesterol; LDL: Low density lipoprotein; ALB: Serum albumin; UTP:24-hUTP; Aetiology: primary kidney disease, including diabetic kidney disease and non-diabetic kidney disease. (*p < 0.05, **p < 0.01, ***p < 0.001,****p < 0.0001.).

For the isolated NR and NS groups, the relationships between urine biomarkers and proteinuria were investigated independently; the findings are displayed in **Figure 3**. In both groups, uAMG and uTRF were positively correlated with proteinuria (isolated NR group: r=0.34, p<0.01 for uAMG and r = 0.62, p < 0.01 for uTRF; NS group: r = 0.40, p < 0.01; and r = 0.42, p < 0.01, respectively).In the isolated NR group, uRBP was positively correlated with proteinuria, with a correlation coefficient of r = 0.45, p < 0.01; whereas uNAG showed no significant correlation. In contrast, within the NS group, uNAG was positively correlated with proteinuria, while no correlation was observed with uRBP (P = 0.673). To compare the differences in the correlation between urinary biomarkers and UTP between the two groups, we performed Fisher’s r-to-z transformation tests. The results showed that the difference in the correlation between uRBP and UTP between the two groups was significant (p = 0.02).

**Figure 3:**
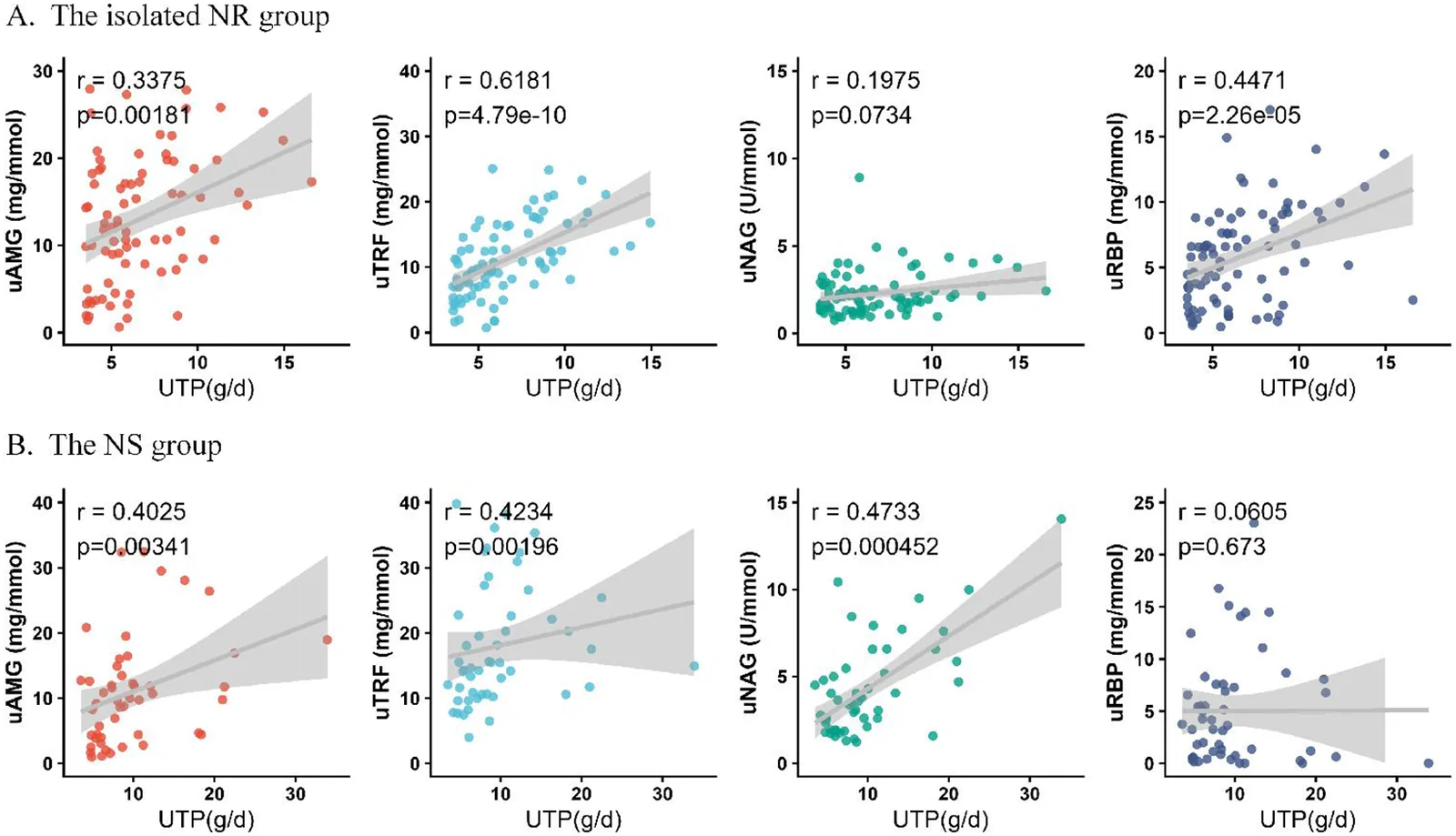
Correlation between urinary biomarkers and proteinuria in isolated NR and NS groups. **(A)** The isolated NR group (n=83). uRBP, uTRF, and uAMG were positively correlated with proteinuria, while uNAG showed no significant correlation. **(B)** The NS group (n=51). uNAG, uTRF, and uAMG were positively correlated with proteinuria, whereas there was no correlation between proteinuria and uRBP. UTP:24-hUTP. *p < 0.05.

### 3.4 Urinary biomarkers in multifactorial models

Multivariate logistic regression analysis was performed to identify biomarkers independently associated with the isolated NR phenotype (**Table 2**). In Model 1, adjusted for age and sex, uRBP were positively associated with the isolated NR phenotype, whereas uTRF and uNAG were associated with the NS phenotype. After further adjustment for eGFR, 24-hUTP, LDL, and primary kidney disease, uRBP and uTRF remained independently associated with the phenotypes. In contrast, the associations observed for uNAG in Model 1 was no longer statistically significant.

**Table 2:** Analysis of the relationship between urinary biomarkers and the isolated NR phenotype using multivariate logistic regression.

Parameter	Model 1			Model 2		
	Adjusted OR	95% CI	*P*-value	Adjusted OR	95% CI	*P*-value
uAMG(mg/mmol)	1.064	1.000-1.134	0.052	1.002	0.908-1.105	0.972
uTRF(mg/mmol)	0.898	0.833-0.969	0.006	0.912	0.838-0.992	0.032
uNAG(U/mmol)	0.522	0.281-0.970	0.041	0.682	0.344-1.352	0.257
uRBP(mg/mmol)	1.203	1.050-1.378	0.008	1.220	1.050-1.418	0.010

To evaluate whether urinary biomarkers can improve the ability to distinguish patients with isolated NR, we constructed two logistic regression models. The base model included established clinical covariates: age, sex, eGFR, 24hUTP, LDL, and primary kidney disease. Model 2 included the clinical covariates from the base model (age, sex, eGFR, 24hUTP, LDL, and primary kidney disease) and was further extended by adding all of the four urinary biomarkers simultaneously.

The area under the receiver operating characteristic (ROC) curve (AUC) was used to evaluate each model’s discriminatory performance. Five-fold cross-validation was performed to obtain an unbiased estimate of the AUC and reduce overfitting. The two models’ differences in AUC were compared using the DeLong test.

The base model achieved a cross-validated AUC of 0.787 (95% CI: 0.708-0.867) for discriminating patients with or without hypoalbuminemia. After adding the urinary biomarkers (Model 2), the AUC increased to 0.838 (95% CI: 0.769-0.908). The improvement in discrimination was statistically significant (DeLong’s test, p< 0.05). (See **Figure 4**).

**Figure 4:**
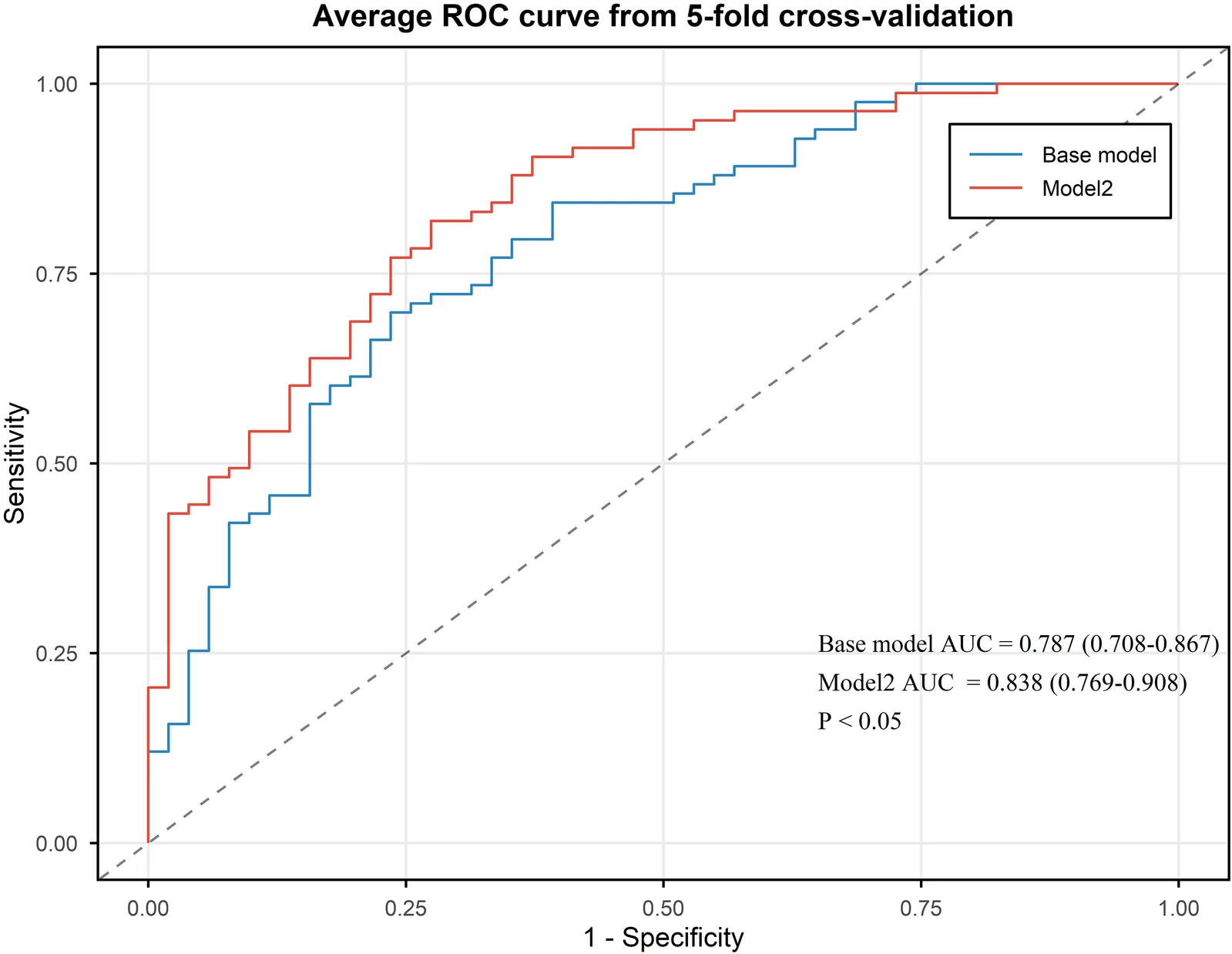
Receiver operating characteristic (ROC) curve for the base model and Model 2. The base model predicted the isolated NR phenotype with an AUC of 0.787 (95% CI: 0.708-0.867). Model 2 predicted the isolated NR phenotype with an AUC of 0.838 (95% CI: 0.769-0.908). The improvement in discrimination was statistically significant (DeLong’s test, p< 0.05).

### 3.5 Differences in urinary biomarkers between DKD subgroups with and without nephrotic syndrome

To assess whether the association between urinary biomarkers and the isolated NR phenotype was influenced by the type of underlying kidney disease, we further examined the interaction between DKD status and urinary biomarkers and conducted exploratory stratified analyses in patients with and without DKD. Of the 134 patients in the overall cohort, 77 had DKD and were included in this subgroup analysis. Of these, 60 cases (77.9%) were classified into the isolated NR group and 17 cases (22.1%) into the NS group.

As shown in **Figure 5**, within the DKD subgroup, the NS group exhibited significantly higher levels of uTRF and uNAG compared to the isolated NR group (p < 0.01), consistent with the findings from the overall CKD cohort. However, in the DKD subgroup, no statistically significant difference in uRBP levels was detected between the isolated NR and NS groups.

**Figure 5:**
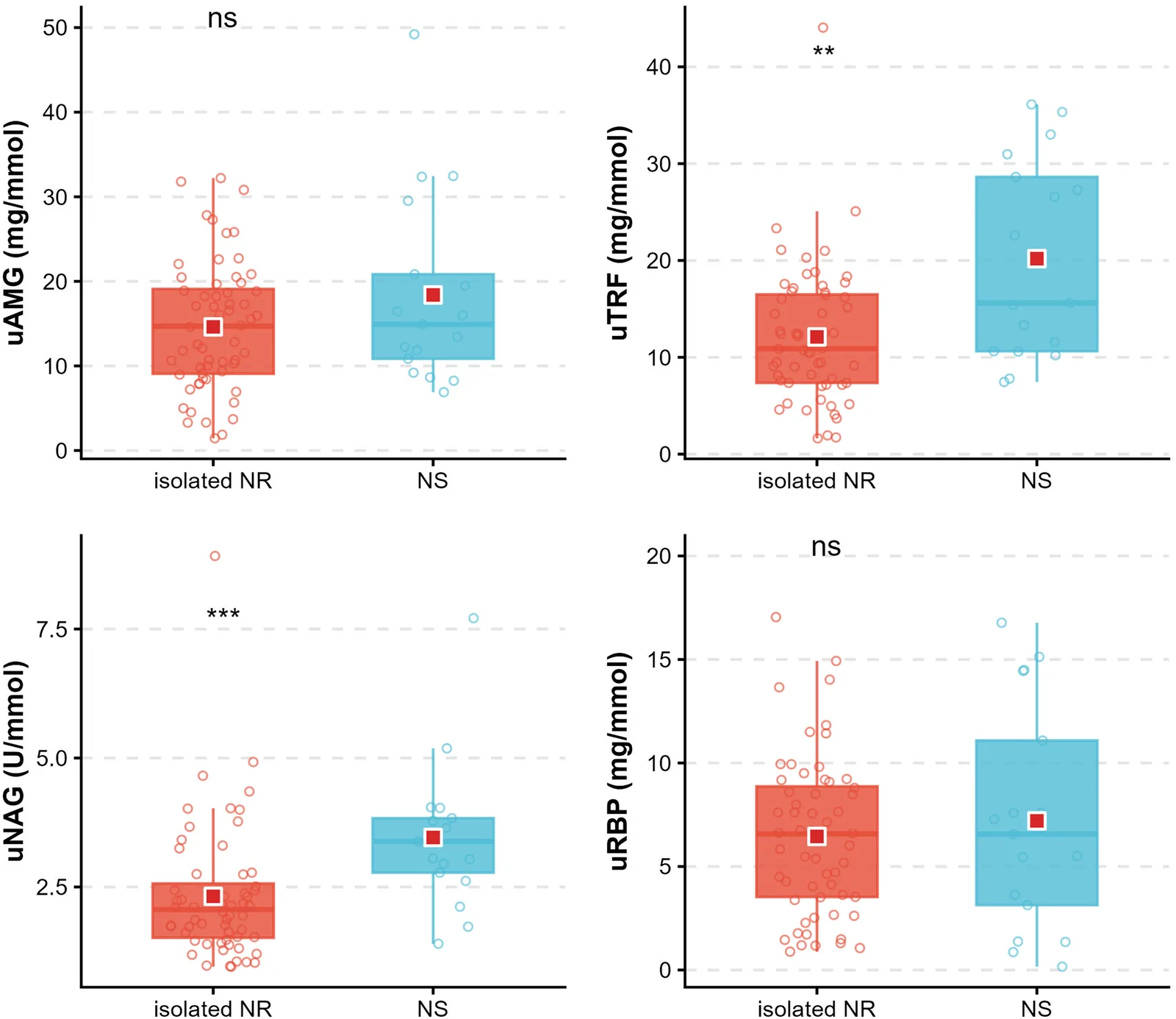
Comparison of urinary biomarkers between DKD subgroups with and without nephrotic hypoalbuminemia. Consistent with the results from the entire CKD cohort, the NS group had considerably greater levels of uTRF and uNAG than the isolated NR group. Nevertheless, there was no statistically significant difference in uRBP levels between the isolated NR and NS groups in the DKD subgroup. Isolated NR: the isolated nephrotic-range proteinuria group; NS: the nephrotic syndrome group; (*p < 0.05, **p < 0.01, ***p < 0.001,****p < 0.0001, ns, *p >* 0.05.).

Subgroup analysis results showed (see **Table 3**) that in the non-DKD group, uRBP was significantly associated with the isolated NR phenotype (OR = 1.31, 95% CI:1.04-1.66, P = 0.02). In contrast, in the DKD group, this association was not statistically significant (OR = 1.18, 95% CI:0.99-1.40, P = 0.06). The test for an interaction between uRBP and DKD status was not statistically significant (interaction P-value =0.793).

**Table 3:** DKD subgroup analysis.

Parameter	DKD			Non-DKD			P for interaction
	Adjusted OR	95% CI	*p*	Adjusted OR	95% CI	*p*	
uAMG(mg/mmol)	0.98	0.88-1.10	0.78	0.97	0.81-1.15	0.70	0.144
uTRF(mg/mmol)	0.92	0.83-1.02	0.11	0.84	0.70-1.01	0.06	0.331
uNAG(U/mmol)	0.66	0.32-1.33	0.24	1.01	0.45-2.29	0.97	0.774
uRBP(mg/mmol)	1.18	0.99-1.40	0.06	1.31	1.04-1.66	0.02	0.793

### 3.6 Sensitivity analyses

As a sensitivity analysis, we stratified the study cohort by eGFR (<30 and ≥30 mL/min/1.73 m²). In both subgroups, Firth-penalized likelihood logistic regression models were constructed; the associations between urinary biomarkers and the isolated NR phenotypes remained consistent in direction, although they did not reach statistical significance—likely due to the reduced sample size within each subgroup (see **Supplementary Table S3** for detailed results).

Since the final model included 10 covariates but there were only 83 events (an average of approximately 8 events per variable), statistical power was limited; we used pre-specified covariates and Firth penalized regression to reduce small-sample bias, and the results were consistent with those of the multivariate regression (see **Supplementary Table S4**).

## 4 Discussion

This study indicates that patients in the isolated NR group and NS group show notable differences in urine biomarker profiles. These findings suggest that, in the setting of NR, the two phenotypes may exhibit different patterns of glomerular-tubular crosstalk.

Proteinuria is not only a key clinical manifestation and biomarker of CKD, but also an independent risk predictor for CKD progression (15). Urinary protein overload can cause dysfunction of renal tubular lysosomes, endoplasmic reticulum stress, and mitochondrial damage. In PTECs, it also activates signaling pathways such as the NLRP3 inflammasome, mitogen-activated protein kinases, and transforming growth factor-β1/Smad, promoting the release of fibrogenic and pro-inflammatory factors (16). Urinary albumin can also trigger the nuclear factor-kappaB signaling pathway and raise mitochondrial reactive oxygen species (ROS) by binding to free fatty acids (FFAs), subsequently aggravating inflammation and apoptosis (17). Human renal biopsy studies have shown that PTECs have the functions of regulating protein reabsorption and influencing proteinuria. Moreover, the degree of proteinuria is significantly correlated with histological indicators such as podocyte injury, interstitial fibrosis and tubular atrophy, supporting that proteinuria is a marker of renal injury as well as a key driver of CKD progression (18). Urinary TRF is a sensitive biomarker of glomerular injury (19). As a lysosomal enzyme in PTECs, NAG has a large molecular weight and cannot pass through the glomerular filtration barrier; therefore, an increase in its urine level reflects tubular injury (20). In this study, the uTRF and uNAG in the NS group increased simultaneously, suggesting more severe damage to the glomerular filtration barrier, inducing urinary protein-related tubular toxicity, and subsequently causing the renal tubules to suffer severe structural and functional damage.

The maintenance of extremely low concentrations of urine protein mainly depends on the endocytosis mediated by megalin and cubilin on the PT. Both can bind to multiple ligands and achieve nearly complete protein reabsorption through an efficient membrane recycling mechanism. Among these RBP is a well-established specific ligand for megalin (21). Additionally, the neonatal Fc receptor (FcRn) can recycle reabsorbed albumin back into the bloodstream through transcellular transport (22). Impaired megalin reabsorption pathways can lead to unique clinical phenotypes. In type 1 Dent disease, megalin at the renal brush border is lost as a result of CLCN5 mutations, thereby causing endocytosis disorders. Clinically, it is characterized by NR without hypoalbuminemia (23, 24). Beyond genetic diseases, recent studies have revealed that megalin expression has a dynamic regulatory mechanism under pathological conditions. In the AKI models, megalin is initially downregulated, which may be a protective response to reduce the uptake of nephrotoxic substances and pro-inflammatory factors. Subsequently, its expression level rises to enhance the reabsorption of potentially protective proteins (25). Histological studies of human proteinuric nephropathy have shown that the expression of megalin in severe proteinuria cases does not increase further compared with mild proteinuria. This may suggest that PTECs reduce intracellular protein load by down-regulating megalin-mediated endocytosis (26). RBP is a low-molecular-weight protein that is mostly reabsorbed by PTECs by receptor-mediated endocytosis after being freely filtered by the glomerulus. The megalin–cubilin system is essential in this process. Therefore, the elevated uRBP levels observed in patients with isolated NR in this study may indicate an abnormality in the PT’s processing of low-molecular-weight proteins. One possible explanation is that, under conditions of sustained protein load, megalin-mediated endocytic function is impaired or undergoes adaptive changes. However, this explanation should currently be regarded only as a mechanistic hypothesis, rather than a proven mechanism. This study did not directly measure megalin/cubilin expression or function in renal tissue, blood, or urine. Furthermore, uRBP is influenced by renal function and underlying kidney disease. The significantly lower eGFR observed in the isolated NR group represents an important potential confounding factor. Therefore, reduced eGFR, differences in etiology, variations in protein filtration load, and alterations in tubular reabsorption capacity may all contribute to the uRBP profile observed in this study. Although other albumin processing pathways, such as FcRn-mediated albumin recycling, may theoretically contribute to serum albumin homeostasis, this study did not evaluate these pathways. Future research should combine renal histopathology, direct detection of megalin and cubilin, urinary protein composition, and indicators of renal tubular albumin processing function to further elucidate the mechanisms underlying the aforementioned different urinary biomarker phenotypes.

The results showing no significant association in the DKD subgroup should be interpreted with caution. Subgroup analysis suggests that the association may vary across different etiological groups, with a stronger effect observed in patients without DKD compared to those with DKD. However, the interaction test did not reach statistical significance, which could indicate a lack of statistical power in the DKD subgroup due to the small sample size. This may also suggest that there is etiological heterogeneity in PT handling of low-molecular-weight proteins among different causes of CKD. Multiple animal studies have shown that in diabetic rat models, the expression of megalin is down-regulated (27, 28). Previous studies have confirmed that early DKD is accompanied by tubular injury before microalbuminuria and glomerular lesions. The injury mechanism involves multiple factors such as hyperglycemia, lipid deposition, inflammation, and oxidative stress (29). This background and homogeneous injury may partially mask the changes in renal tubular reabsorption function caused by differences in proteinuria levels. It is suggested that renal tubular injury in patients with DKD may be more common and occur earlier, and its biomarker spectrum may be different from that of non-DKD patients. In clinical practice, for patients with DKD who present with NR, even if the ALB level is normal, the risk of extensive renal tubular injury that already exists should be highly valued. Therefore, the association between uRBP and the isolated NR phenotype in the general population should not be interpreted as reflecting a uniform mechanism applicable to all causes of CKD. The findings of this study should be considered exploratory; future studies with larger sample sizes are needed to determine whether the etiology of the disease does indeed alter this association. Furthermore, incorporating biomarkers that reflect injury along different pathophysiological pathways will be essential for clarifying the complex mechanisms in this population.

In conclusion, this study indicates that patients with NR with and without hypoalbuminemia exhibit distinct urine biomarker profiles, suggesting that there may be differences in the mechanisms of glomerular-tubular injury. The isolated NR phenotype may be associated with changes in the processing of low-molecular-weight proteins by the PTECs, whereas the NS phenotype likely results from combined glomerular and tubular injury driven by severe glomerular barrier dysfunction. This provides insights into the mechanisms underlying the clinical heterogeneity of NR, laying a theoretical foundation for future intervention studies targeting the megalin pathway. Additionally, it also provides a basis for identifying different clinical phenotypes of CKD and implementing precision management.

This study had a comparatively modest total sample size and was a single-center retrospective cross-sectional study. The influence of residual confounding factors cannot be ruled out despite multivariate adjustment. This was a retrospective study; all laboratory and clinical data were extracted from medical records. Therefore, detailed information regarding sample collection and handling, concomitant medications, and factors such as inflammation, nutritional status, and fluid status could not be fully obtained. These unmeasured factors may have influenced our study results. This study did not directly measure the expression or function of megalin and cubilin in renal tissue, blood, or urine; therefore, the interpretation that receptor-mediated protein processing pathways in the PT are involved remains a mechanistic hypothesis. Furthermore, this study lacks detailed data on the composition of urinary proteins. Although 24-hUTP was quantified, urinary albumin excretion, the urinary albumin-to-total protein ratio, urinary protein electrophoretic components, and the protein selectivity index were not systematically assessed. Therefore, it is currently impossible to determine whether the elevated uRBP levels observed in patients without hypoalbuminemia truly reflect an increased relative proportion of low-molecular-weight proteins in total urinary protein excretion. Future studies will need to combine quantitative analysis of urinary protein composition with direct evaluation of megalin–cubilin-mediated endocytosis to further clarify this issue.

## Data Availability

The raw data supporting the conclusions of this article will be made available by the authors, without undue reservation.
