# Supplementary material for: Differences in urinary biomarker profiles in patients with nephrotic-range proteinuria with and without hypoalbuminemia: a single-center cross-sectional study
Source: Front Endocrinol (Lausanne). 2026 Oct 2;17:1938326. doi: 10.3389/fendo.2026.1938326 (PMC13671992; doi:10.3389/fendo.2026.1938326)
Supplement: Supplementary file 1 [file SupplementaryFile1.docx]

Supplementary Materials

1. **Statistical Analysis**

R software version 4.5.2 was used for statistical analysis. *P*-values below 0.05 were deemed statistically significant. To determine normality, the Shapiro-Wilk test was employed. The mean ± standard deviation (SD) is the expression for continuous data that follows a normal distribution. In contrast, the median with an interquartile range (IQR) is used to represent those with a non-normal distribution. Categorical data are described using counts (percentages). For regularly distributed data, the independent samples t-test was employed to compare continuous variables between two groups; for non-normally distributed data, the Wilcoxon rank-sum test was employed. To compare categorical variables between groups, the chi-square test was employed. The associations between urine biomarkers and other clinical parameters were evaluated using Spearman correlation analysis. In addition, we use the Benjamini–Hochberg method to control the false discovery rate (FDR).The relationship between urine biomarkers and the isolated NR phenotype was investigated using multivariate logistic regression models. We included all four biomarkers simultaneously in multivariate logistic regression models. This connection was corrected for age and sex in Model 1; in Model 2, it was corrected for sex, age, eGFR, 24-hUTP, LDL, and primary kidney disease. Multicollinearity was assessed using the variance inflation factor. The number and proportion of missing values for each urinary biomarker were reported separately. Assuming that missing values were random, the chained equation multiple interpolation method was used to interpolate the missing data, with a total of 10 imputations (m=10). The interpolation model included demographic characteristics, renal function, proteinuria, serum albumin, blood lipids, underlying kidney disease, and other available urinary biomarkers. Rubin’s rule was used to combine the estimates from each imputed dataset, and a complete-cases analysis was further conducted as a sensitivity analysis. Urine biomarker data are displayed as the ratio of concentration to matching urine creatinine levels in order to normalize the effects of urine concentration.We conducted sensitivity analyses to assess the robustness of the study results. First, we stratified the study cohort based on CKD staging (eGFR ≥ 30 vs. < 30 mL/min/1.73 m²) and performed Firth-penalized likelihood logistic regression analyses within each stratum to evaluate whether the association between urinary biomarkers and the isolated NR phenotype varied with changes in renal function. Second, we used a Firth-penalized likelihood logistic regression model based on imputed datasets to address potential small-sample bias.

Variables with missing values included uNAG (9.0%)(See Supplementary Figure S1). In the isolated NR group, there were 10 missing values, and in the nephrotic syndrome (NS) group, there were 3 missing values.

**Supplementary Figure S1.** Number of missing

**Supplementary Figure S1.** Number of missing. The total sample size was 134 cases. Variables with missing values included uNAG (9.0%). There are no missing values for the other variables.

Hb: Hemoglobin; Scr: Serum Creatinine; eGFR: Estimated glomerular filtration rate; UREA: urea; UA: Uric acid; TC: Total cholesterol; LDL: Low density lipoprotein; ALB: Serum albumin; UTP:24-hUTP; UCr: Urine Creatinine; Aetiology: primary kidney disease, including diabetic kidney disease and non-daibetic kidney disease.AMGUcr: urinary α1-microglobulin; TRFUcr: urinary transferrin; RBPUcr: urinary retinol binding protein; NAG: urinary N-acetyl-β-D-glucosaminidase;

2. **Standardized differences**

**Supplementary Tabel S1** Baseline Characteristic Comparison of CKD Patients and Standardized mean difference

| characteristic | Overall（n=134) | isolated NR(n=83) | NS(n=51) | *P* value | SMD |
| --- | --- | --- | --- | --- | --- |
| Male sex(%) | 96 (71.64) | 68 (81.93) | 28 (54.90) | 0.002 | 0.608 |
| Age(mean (SD))，yr | 54.16 (14.18) | 54.66 (13.01) | 53.35 (16.02) | 0.606 | 0.090 |
| Hemoglobin(mean (SD))，g/L | 115.52 (21.62) | 114.47 (20.78) | 117.24 (23.03) | 0.474 | 0.126 |
| Creatinine(median [IQR])，μmol/L | 244.55 [103.82, 357.68] | 282.30 [134.35, 449.55] | 141.80 [88.40, 244.55] | <0.001 | 0.697 |
| eGFR (median [IQR]),mL/min/1.73 m^2^ | 26.47 [16.28, 67.75] | 20.69 [11.86, 47.32] | 45.88 [21.43, 82.54] | <0.001 | 0.626 |
| Urea(median [IQR])，mmol/L | 13.89 [7.87, 20.49] | 16.55 [9.81, 23.74] | 10.25 [6.57, 15.13] | <0.001 | 0.715 |
| Uric acid(mean (SD))，μmol/L | 407.97 (98.98) | 420.08 (100.55) | 388.26 (94.02) | 0.071 | 0.327 |
| Serum albumin(median [IQR])，g/L | 31.80 [27.33, 36.98] | 35.60 [33.00, 38.45] | 25.50 [22.00, 28.25] | <0.001 | 2.823 |
| 24hUTP(median [IQR])，mg | 6504.00 [4871.75, 9227.75] | 5862.00 [4453.00, 8517.50] | 8338.00 [6010.00, 11268.50] | <0.001 | 0.701 |
| Total cholesterol  (median [IQR])，mmol/L | 4.94 [4.07, 6.53] | 4.40 [3.65, 5.36] | 6.16 [4.97, 7.91] | <0.001 | 0.969 |
| Low density lipoprotein  (median [IQR])，mmol/L | 2.80 [2.23, 4.12] | 2.41 [1.90, 3.13] | 3.73 [2.71, 5.10] | <0.001 | 0.960 |
| **Primary disease(%)** |  |  |  |  |  |
| Diabetic kidney disease | 77 (57.46) | 60 (72.29) | 17 (33.33) | <0.001 | 0.912 |
| Chronic glomerulonephritis | 46 (34.33) | 16 (19.28) | 30 (58.82) |  |  |
| Other or unknown | 11 ( 8.21) | 7 ( 8.43) | 4 ( 7.84) |  |  |

**SMD:** Standardized mean difference. SMD < 0.1 indicates a negligible difference between groups. *SMD > 0.1 indicates potential imbalance requiring adjustment in subsequent analyses.

3. **Sensitivity analyses**
   3.1 **Complete-case analyse**

**Supplementary Tabel S2** Multivariate Logistic Regression in the complete case

| Parameter | Model 1 | | | Model 2 | | |
| --- | --- | --- | --- | --- | --- | --- |
|  | Adjusted OR | 95% CI | *p*-Value | Adjusted OR | 95% CI | *p*-Value |
| uAMG(mg/mmol) | 1.060 | 0.993-1.137 | 0.086 | 1.019 | 0.919-1.129 | 0.721 |
| uTRF(mg/mmol) | 0.913 | 0.838-0.981 | 0.020 | 0.931 | 0.845-1.013 | 0.116 |
| uNAG(U/mmol) | 0.392 | 0.230-0.607 | 0.000 | 0.484 | 0.257-0.831 | 0.015 |
| uRBP(mg/mmol) | 1.229 | 1.064-1.438 | 0.007 | 1.254 | 1.072-1.498 | 0.007 |

**Supplementary Tabel S2** Multivariate Logistic Regression in the complete case. The multivariable logistic regression results from the imputed datasets were consistent with those from the complete-case analysis in terms of effect direction.

3.2 **eGFR stratification analysis**

**Supplementary Tabel S3** Firth-penalized likelihood logistic regression in the eGFR ≥30 and eGFR <30mL/min/1.73 m² subgroups.

| Parameter | eGFR≥30 | | | eGFR<30 | | |
| --- | --- | --- | --- | --- | --- | --- |
|  | Adjusted OR | 95% CI | *p*-Value | Adjusted OR | 95% CI | *p*-Value |
| uAMG(mg/mmol) | 0.99 | 0.83-1.17 | 0.89 | 1.02 | 0.92-1.13 | 0.71 |
| uTRF(mg/mmol) | 0.89 | 0.79-1.01 | 0.06 | 0.94 | 0.85-1.03 | 0.19 |
| uNAG(U/mmol) | 0.99 | 0.56-1.76 | 0.97 | 0.64 | 0.30-1.33 | 0.23 |
| uRBP(mg/mmol) | 1.17 | 0.98-1.39 | 0.08 | 1.16 | 0.96-1.41 | 0.13 |

**Supplementary Tabel S3** Firth-penalized likelihood logistic regression in the eGFR ≥30 and eGFR <30mL/min/1.73 m² subgroups. In both subgroups, Firth-penalized likelihood logistic regression models were constructed; the associations between urinary biomarkers and the isolated NR phenotypes remained consistent in direction, although they did not reach statistical significance.

3.3 **Firth-penalized likelihood logistic regression**

**Supplementary Tabel S4** Firth-penalized likelihood logistic regression using the imputed datasets.

| Parameter | Model 1 | | | Model 2 | | |
| --- | --- | --- | --- | --- | --- | --- |
|  | Adjusted OR | 95% CI | *p*-Value | Adjusted OR | 95% CI | *p*-Value |
| uAMG(mg/mmol) | 1.06 | 1.00-1.12 | 0.05 | 1.00 | 0.92-1.09 | 0.95 |
| uTRF(mg/mmol) | 0.91 | 0.85-0.97 | 0.00 | 0.92 | 0.86-0.99 | 0.03 |
| uNAG(U/mmol) | 0.56 | 0.33-0.93 | 0.03 | 0.73 | 0.43-1.25 | 0.25 |
| uRBP(mg/mmol) | 1.18 | 1.05-1.34 | 0.01 | 1.19 | 1.04-1.35 | 0.01 |

**Supplementary Tabel S4** Firth-penalized likelihood logistic regression using the imputed datasets. The results were consistent with those of the multivariate regression.

3.4 **Multicollinearity Test**

**Supplementary Tabel S5** Multicollinearity Test for Model 2 based on imputed datasets.

| Parameter | Average VIF | Maximum VIF |
| --- | --- | --- |
| Sex | 1.30 | 1.33 |
| Age | 1.22 | 1.24 |
| eGFR | 2.56 | 2.66 |
| UTP | 1.89 | 1.94 |
| LDL | 1.35 | 1.37 |
| Aetiology | 1.33 | 1.35 |
| AMGUcr | 2.96 | 3.11 |
| TRFUcr | 1.89 | 1.99 |
| NAGUcr | 1.76 | 1.93 |
| RBPUcr | 1.95 | 2.06 |

**Supplementary Tabel S5** Multicollinearity Test for Model 2 based on imputed datasets. Multicollinearity was assessed using the variance inflation factor, and all values were below 5.
